# Identification and validation of glycolysis-related diagnostic signatures in diabetic nephropathy: a study based on integrative machine learning and single-cell sequence

**DOI:** 10.3389/fimmu.2024.1427626

**Published:** 2025-01-23

**Authors:** Xiaoyin Wu, Buyu Guo, Xingyu Chang, Yuxuan Yang, Qianqian Liu, Jiahui Liu, Yichen Yang, Kang Zhang, Yumei Ma, Songbo Fu

**Affiliations:** ^1^ School of Basic Medical Sciences, Lanzhou University, Lanzhou, China; ^2^ The First Clinical Medical College, Lanzhou University, Lanzhou, China; ^3^ Department of Endocrinology, First Hospital of Lanzhou University, Lanzhou, China; ^4^ Obstetrics and Gynecology Hospital, Fudan University, Shanghai, China; ^5^ Shanghai Key Laboratory Female Reproductive Endocrine-Related Diseases, Shanghai, China; ^6^ Xifeng District People’s Hospital, Qingyang, China; ^7^ Qilihe District People’s Hospital, Lanzhou, China; ^8^ Gansu Provincial Endocrine Disease Clinical Medicine Research Center, Lanzhou, China

**Keywords:** diabetic nephropathy, glycolysis, machine learning, diagnostic signatures, single cell

## Abstract

**Background:**

Diabetic nephropathy (DN) is a complication of systemic microvascular disease in diabetes mellitus. Abnormal glycolysis has emerged as a potential factor for chronic renal dysfunction in DN. The current lack of reliable predictive biomarkers hinders early diagnosis and personalized therapy.

**Methods:**

Transcriptomic profiles of DN samples and controls were extracted from GEO databases. Differentially expressed genes (DEGs) and their functional enrichments were identified. Glycolysis-related genes (GRGs) were selected by combining DEGs, weighted gene co-expression network, and glycolysis candidate genes. We established a diagnostic signature termed GScore via integrative machine learning framework. The diagnostic efficacy was evaluated by decision curve and calibration curve. Single-cell RNA sequence data was used to identify cell subtypes and interactive signals. The cMAP database was used to find potential therapeutic agents targeting GScore for DN. The expression levels of diagnostic signatures were verified *in vitro*.

**Results:**

Through the 108 combinations of machine learning algorithms, we selected 12 diagnostic signatures, including CD163, CYBB, ELF3, FCN1, PROM1, GPR65, LCN2, LTF, S100A4, SOX4, TGFB1 and TNFAIP8. Based on them, an integrative model named GScore was established for predicting DN onset and stratifying clinical risk. We observed distinct biological characteristics and immunological microenvironment states between the high-risk and low-risk groups. GScore was significantly associated with neutrophils and non-classical monocytes. Potential agents including esmolol, estradiol, ganciclovir, and felbamate, targeting the 12 diagnostic signatures were identified. *In vitro*, ELF3, LCN2 and CD163 were induced in high glucose-induced HK-2 cell lines.

**Conclusion:**

An integrative machine learning frame established a novel diagnostic signature using glycolysis-related genes. This study provides a new direction for the early diagnosis and treatment of DN.

## 1 Introduction

Diabetic nephropathy (DN) is a prevalent and severe microvascular complication of diabetes mellitus (DM), with epidemiological studies estimating the risk of developing DN to be as high as 25-40% (1). As a leading cause of end-stage renal disease globally and a precursor for chronic kidney disease, DN imposes a considerable healthcare burden on patients, families, and society (2). A comprehensive understanding of the pathogenesis and biological characteristics of DN, along with the identification of reliable predictive biomarkers for high-risk groups, is essential for early prevention and detection or for slowing disease progression. Currently, clinical practice lacks effective early diagnostic markers, resulting in many diabetic patients unknowingly progressing to DN due to inadequate monitoring.

Glycolysis, a pivotal metabolic pathway ubiquitously present in the cells of all living organisms, is responsible for the conversion of glucose into pyruvate (3). This process, occurring in the cytoplasm, stands at the heart of energy metabolism, essential for meeting the bioenergetic demands of the cell. In DN, the impaired utilization of glucose by cells leads to changes in the bioactivity of glycolysis (4). This metabolic reprogramming not only affects the function of kidney cells but may also exacerbate kidney damage. In addition, glycolysis in DN may interact with pathological processes such as inflammation (5), oxidative stress (6), and extracellular matrix accumulation (7), which are key factors in the progression of DN. Therefore, a deeper understanding of the mechanism of glycolysis in DN may lead to the discovery of new predictive or intervention targets, providing a theoretical basis for new treatment strategies.

**Figure 1:**
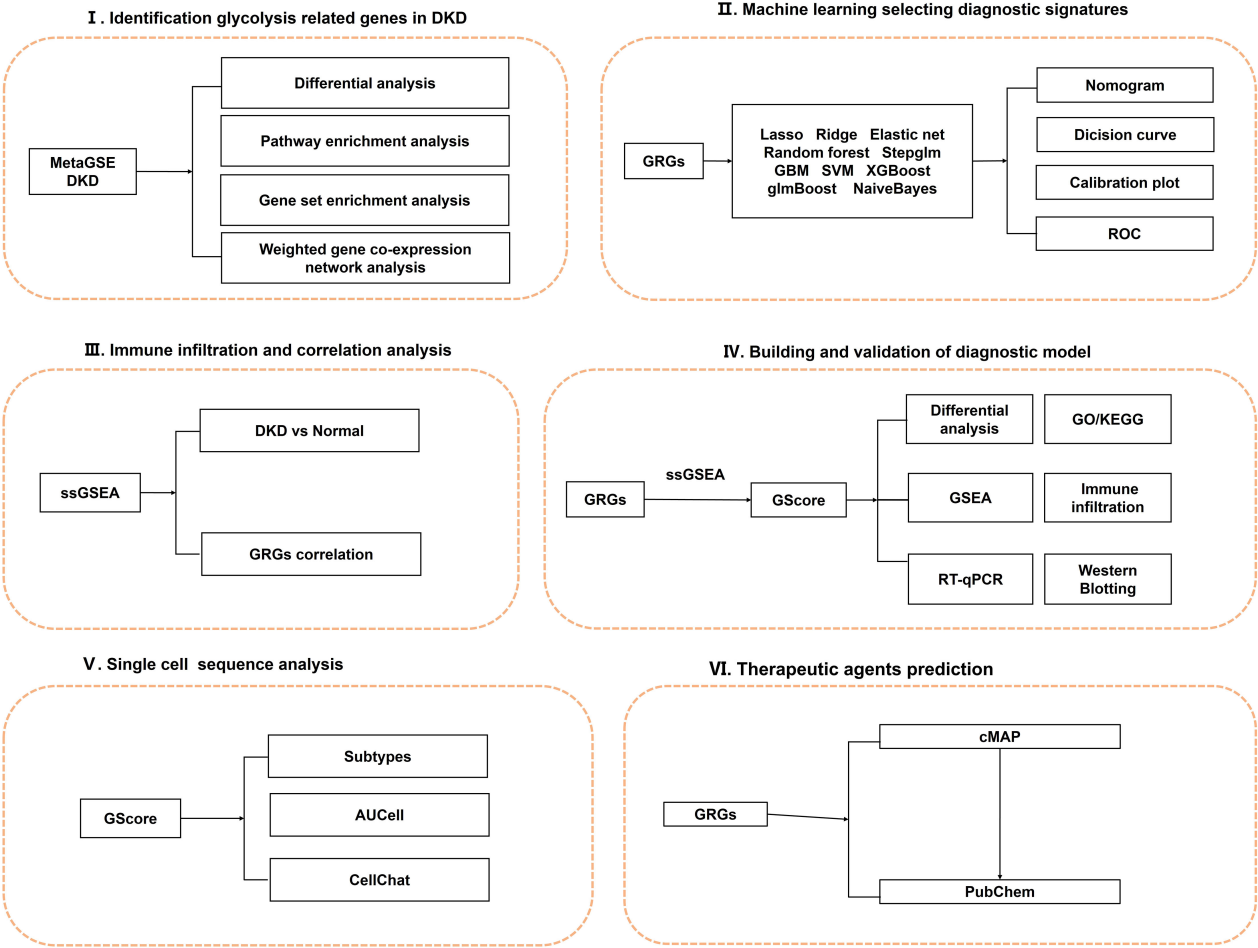
The overall design of this study.

In this study, we applied 108 combinations of 10 machine learning algorithms, including Elastic Net (Enet), Random Forest (RF), and Support Vector Machine (SVM), to identify predictive signatures associated with glycolysis in DN. Utilizing these genes, we developed a diagnostic prediction system termed GScore. The diagnostic model demonstrated satisfactory predictive performance and stability across the training/internal validation set, and three independent external validation sets. We conducted a comprehensive analysis of GScore regarding immune infiltration, pathway enrichment, therapeutic drugs, and microenvironment characteristics at single-cell resolution. Furthermore, *in vitro* experiments indicated significant differences in the expression levels of the 12 diagnostic signatures. Our findings offer a novel perspective on the aberrant glycolysis state during DN progression and highlight potential avenues for early diagnosis and treatment strategies. The overall design of this study is illustrated in **Figure 1**.

## 2 Materials and methods

### 2.1 Data acquisition and preprocessing

We obtained gene expression matrix from the Gene Expression Omnibus (GEO) database (http://www.ncbi.nlm.nih.gov/geo). The data collection criteria included (1): expression profiling by array, (2) glomerular or tubular tissue, (3) homo sapiens. This study incorporated GSE47183 (8), GSE47184 (8), GSE104948 (9), GSE104954 (9), GSE96804 (10), GSE30528 (11), and GSE30529 (11) from microarrays, and GSE183276 (12) from single-cell RNA sequence. Detailed information on each dataset is provided in **Supplementary Table 1**. Only genes present in all datasets were retained. To filter diagnostic signatures and assess their predictive performance for DN occurrence, we combined GSE47183, GSE47184, GSE104948, and GSE104954 into a meta-cohort named MetaGSE, which contained 56 DN samples and 36 normal controls. The “normalizeBetweenArrays” function from the “limma” package was utilized for data correction (13). Subsequently, “removeBatchEffect” function was employed to eliminate batch effect across datasets (13). Principal component analysis (PCA) and t-Distributed Stochastic Neighbor Embedding (t-SNE) were utilized to assess data distribution (**Supplementary Figure 1**).

### 2.2 Differentially expressed genes identification and functional enrichment

Differentially expressed genes (DEGs) between DN and control groups were identified using limma package (13) in the MetaGSE dataset. The thresholds were adjusted P < 0.05 and |log2FC| > 0.5. The DEGs results are detailed in **Supplementary Table 2.** Visualization was conducted using the “ggplot2” and “complexheatmap” R packages. To elucidate biological function of DEGs, we conducted Gene Ontology (GO) (14), Kyoto Encyclopedia of Genes and Genomes (KEGG) (15), and REACTOME (16) enrichment analysis by the “clusterProfiler” package (17). Results were deemed statistically significant with Benjamini-Hochberg adjusted p-values < 0.05.

### 2.3 Pathway enrichment

The Gene set “h.all.v2023.2.Hs.symbols” from the Molecular Signatures Database (MsigDB; https://www.gsea-msigdb.org/gsea/msigdb) (18) was utilized for Gene Set Enrichment Analysis (GSEA) (19) to evaluate potential biological processes. The normalized enrichment score (NES) and false discovery rate (FDR) were used to quantify enrichment magnitude and assess statistical significance.

### 2.4 Gene correlation analysis

Pearson correlation analysis was performed on DEGs from both DN and controls to evaluate distinct gene expression patterns.

### 2.5 Weighted gene co-expression network construction and hub genes identification

To identify gene sets with highly correlated expression changes and screen for candidate biomarkers associated with DN, we employed Weighted gene co-expression network analysis (WGCNA) (20). Initially, hierarchical clustering was conducted to eliminate outliers. An appropriate soft power was then selected to construct a weighted adjacency matrix, which was transformed into a topological overlap matrix (TOM) characterized by distinct colors and module eigengenes (MEs). Each module contained a minimum of 50 genes, and adjacent modules with a similarity threshold of at least 0.7 were merged. Finally, Pearson’s correlation coefficient was calculated to assess the correlation between each ME and the DN samples.

### 2.6 Identification of the glycolysis-related genes

We extracted non-redundant glycolysis-related candidate genes from the relevant published literature, Genecards (https://www.genecards.org/), as well as from the HALLMAR_GLYCOLYSIS and REACTOME_GLYCOLYSIS gene sets within the MsigDB. Additionally, we identified hub genes within the module highly correlated with DN by applying a Gene Significance (GS) threshold greater than 0.25 and a Module Membership (MM) threshold greater than 0.7. Ultimately, we identified glycolysis-related genes (GRGs) by overlapping the hub genes, glycolysis-related candidate genes, and DEGs.

### 2.7 Construction of the glycolysis-related genes score by integrative machine learning models

To develop a diagnostic model with high predictive accuracy, we followed these steps (1): The MetaGSE cohort was randomly divided into an training set and an internal validation set at a ratio of 7:3. The individual external validation sets were GSE96804, GSE30528, and GSE30529. We employed several integrative machine learning algorithms, including Lasso, Ridge, Elastic Net (Enet), Random Forest (RF), Stepglm, Generalized Boosted Regression Modeling (GBM), Support Vector Machine (SVM), Extreme Gradient Boosting (XGBoost), glmBoost, and Naive Bayes. Combinations of these 10 algorithms were applied to the MetaGSE for signature selection and model construction using 10-fold cross-validation. (2) All models were evaluated on both the internal training cohort and the external validation cohorts. (3) We calculated the Area Under the Curve (AUC) and 95% confidence intervals (Cis) for MetaGSE, GSE96804, GSE30528, and GSE30529, subsequently ranking all models based on the AUC index. (4) The top 12 potential diagnostic signatures identified by the most efficient algorithm combinations were CD163, CYBB, ELF3, FCN1, GPR65, LCN2, LTF, PROM1, S100A4, SOX4, TGFBI, and TNFAIP8. (5) Based on these selected signatures, we established a diagnostic model predictive of DN occurrence, termed the GScore. The GScore is derived from single-sample Gene Set Enrichment Analysis (ssGSEA) using the “GSVA” R package: GScore = ssGSEAsocre (12 diagnostic signatures).

### 2.8 Evaluation of GScore by multiple approaches

We first developed a nomogram based on diagnostic signatures to quantify the risk of DN (21). We then assessed the predictive ability and diagnostic accuracy of both the nomogram and the GScore using decision curve analysis and calibration curves (22). Additionally, we constructed Receiver Operating Characteristic (ROC) curves for each diagnostic signature across the training and validation sets.

### 2.9 Immune cell infiltration assessment and correlation analysis

To investigate the immunological characteristics between DN samples and controls, we employed the ssGSEA method to quantify the infiltration levels of 28 immune cell types and assess correlations among these cells (23). Additionally, we utilized the Spearman rank correlation test to evaluate the relationship between each diagnostic signature and immune cells in the DN samples.

### 2.10 Risk evaluation and biological characteristic analysis based on GScore

To further investigate the stratified predictive ability of the GScore, we calculated it for all samples. Based on the median GScore, subjects were classified into high-risk and low-risk groups. We then aimed to elucidate the distinct characteristics between these groups by examining the expression patterns of GRGs and DEGs. Additionally, we conducted GO (14), KEGG (15), GSEA (19) enrichment analysis. Moreover, we assessed the correlation between the GScore and the infiltration levels of various immune cells (24).

### 2.11 Single-cell RNA-seq data collection and processing

We collected single-cell RNA sequencing data of 14 DN samples and 20 normal controls in the GSE183276. Seurat (version 4.4.0) (25) was used for the analysis of the single-cell data. Cells with mitochondrial gene expression below 50% and those containing at least three cells with between 500 and 5000 unique multiplex indices (UMIs) were retained. We then selected 3000 highly variable genes for downstream analyses. Harmony (26) was employed to correct for batch effects across samples. Cell clusters were identified using the “FindNeighbors” and “FindClusters” functions, and visualized with t-SNE. Concurrently, we calculated differentially expressed genes within each cell cluster using the “FindAllMarkers” function and the Wilcoxon test. Major cell types were annotated based on established markers. To evaluate the GScore for each cell type, we utilized AUCell (27) to assess the bioactivity of GRGs in each celltypes and map the GScore. Finally, we performed cell interaction analysis on the subtypes with the highest GScore using CellChat (28).

### 2.12 Potential therapeutic agents prediction

To further explore the clinical implications of GRGs, we examined the Connectivity Map database (cMAP; https://clue.io/) (29) to identify small molecule agents with potential therapeutic effects. The 3D structures of these small molecules were obtained from pubchem (https://pubchem.ncbi.nlm.nih.gov/) (30).

### 2.13 Cell culture and establishment of *in vitro* model of DN

The proximal tubular epithelial cell line (HK-2) was purchased from FuHeng Biology and cultured in DMEM/F12 (Servicebio, China) with 10% fetal bovine serum (FBS, Pricella, China) and 1% penicillin-streptomycin (Biosharp, China) at 37°CC in a humidified atmosphere of 5% CO2. HK2 cells were inoculated into 96-well plates with the number of 5×10^3 per well. After 24 hours of serum-free culture, the culture medium with glucose concentration of 17.5mM, 30mM, 45mM and 60mM was used for 48h. Then, according to the scheme given by the reagent manufacturer, the Cell Counting Kit-8(Beyotime, C0038, China) was used to determine the cell viability, and the high glucose concentration at 80% of the cell viability was taken as the glucose concentration of the diabetic nephropathy model *in vitro*. HK-2 cells were inoculated in 6-well plates and cultured in serum-free medium for 24 h, then exposed to serum-free medium supplemented with D-glucose (HG) for 48 h. The cells cultured with 17.5mM D-glucose (NG) were used as control and treated for 48 h.

### 2.14 RNA extraction and qRT-PCR

Total RNA from HK-2 were extracted using the SteadyPure Universal RNA Extraction KitII (Accurate Biotechnology, AG21022, China). The ABScript Neo RT Master Mix for qPCR with gDNA Remover (ABclonal, RK20433, China) was used for the reverse transcription of miRNA. 2X Universal SYBR Green Fast qPCR Mix ((ABclonal, RK21203, China) was utilized for conducting real-time quantitative PCR. The relative expression levels were calculated using two-delta delta CT. Specially designed primers are provided in **Table 1.**

**Table 1:** Primers used for RT-qPCR.

Gene	Forward primer sequence(5’-3’)	Reverse primer sequence(5’-3’)
SOX4	AATGCCGAGAACACGGAAG	ACCACACCATGAAGGCGTTC
TGFBI	CTTCGCCCCTAGCAACGAG	TGAGGGTCATGCCGTGTTTC
TNFAIP8	AAGATGAGCTAGCATTGATGGAG	TTCATTTAACAGCCTGGATAACAC
S100A4	GCCCTGGATGTGATGGTGTC	GTTGCTGTCCAAGTTGCTCATC
PROM1	GGAGTCGGAAACTGGCAGATAG	TGAACGCCTTGTCCTTGGTAG
LTF	TGCCCAACAGCAACGAGAG	TCCATCAGTGTTCTGCAAGACAG
LCN2	CAGGGGAAGTGGTATGTGGTAG	TGGCAACCTGGAACAAAAGTC
FCN1	CATTCAAGGTGGCTGACGAG	CATCATTGTCTTGGTCTTTGGTG
ELF3	CAGATGTCATTGGAGGGTACAGA	CGCTTGCGTCGTACTTGTTC
CYBB	TCGCATCCATTCTCAAGTCAG	GCATTGTTCCTTTCCTGCATC
CD163	TTTGTCAACTTGAGTCCCTTCAC	TCCCGCTACACTTGTTTTCAC
GPR65	GAAATGGCAAATCAACCTCAAC	CTTGTTTTCCGTGGCTTTATTG
β-actin	TGGCACCCAGCACAATGAA	CTAAGTCATAGTCCGCCTAGAAGCA

### 2.15 Western blotting

Radio immunoprecipitation assay buffer (EpiZyme, PC101, China) was used to extract cellular proteins. The protein concentration was quantified via a bicinchoninic acid (BCA) protein assay kit (EpiZyme, ZJ102, China). Then, all the protein samples were mixed with 5X SDS-PAGE protein loading buffer (EpiZyme, LT103, China). After all the samples were denatured at 100°C for 10 min. Separation via SDS-PAGE, the protein was shifted to PVDF membranes(EpiZyme, WJ002, China) and seal the film with 5% skim milk powder for 1 h. Afterward, the membranes were incubated overnight with primary antibodies CD163 (1:5000, Proteintech, 16646-1-AP, China) and β-actin(1:10000, Proteintech, 66009-1-IG, China). Then incubated with HRP conjugated antibody at room temperature for 1 h. Bands were detected by enhanced chemiluminescent(EpiZyme, SQ203, China) and analyzed with ImageJ 1.54g software (NIH, USA) based on the gray value of target protein(CD163) and gray ratio of the internal reference (β-actin).

### 2.16 Statistical analysis

All statistical analyses were performed using R software (version 4.2.2, https://www.r-project.org/). Pearson correlation was used to analyze two continuous variables, while comparisons between two groups were performed using the t-test. Correlation analysis between genes and immune cells employed the Spearman method, utilizing either the “ggpubr” or “stats” R packages. The Wilcoxon rank-sum test assessed differences between two non-normally distributed variables. Receiver operating characteristic (ROC) curves were generated using the “pROC” R package (31). An adjusted p-value or a p-value with a significance threshold of 0.05 was considered statistically significant.

## 3 Results

### 3.1 Microarray data collection and preprocessing

Four microarray datasets—GSE47183, GSE47184, GSE104948, and GSE104954—were normalized and merged into a large training/internal validation cohort named MetaGSE. The batch effect was corrected by using the “removeBatchEffect” function of “limma” package (13). This MetaGSE contains 56 DN samples and 36 Normal controls. Following normalization, the data distributions from each dataset fell within a similar range (**Supplementary Figures 1A, B**), and the batch effects between datasets were effectively mitigated (**Supplementary Figures 1C, D**).

### 3.2 Identification of DEGs and functional annotations

**Figure 2:**
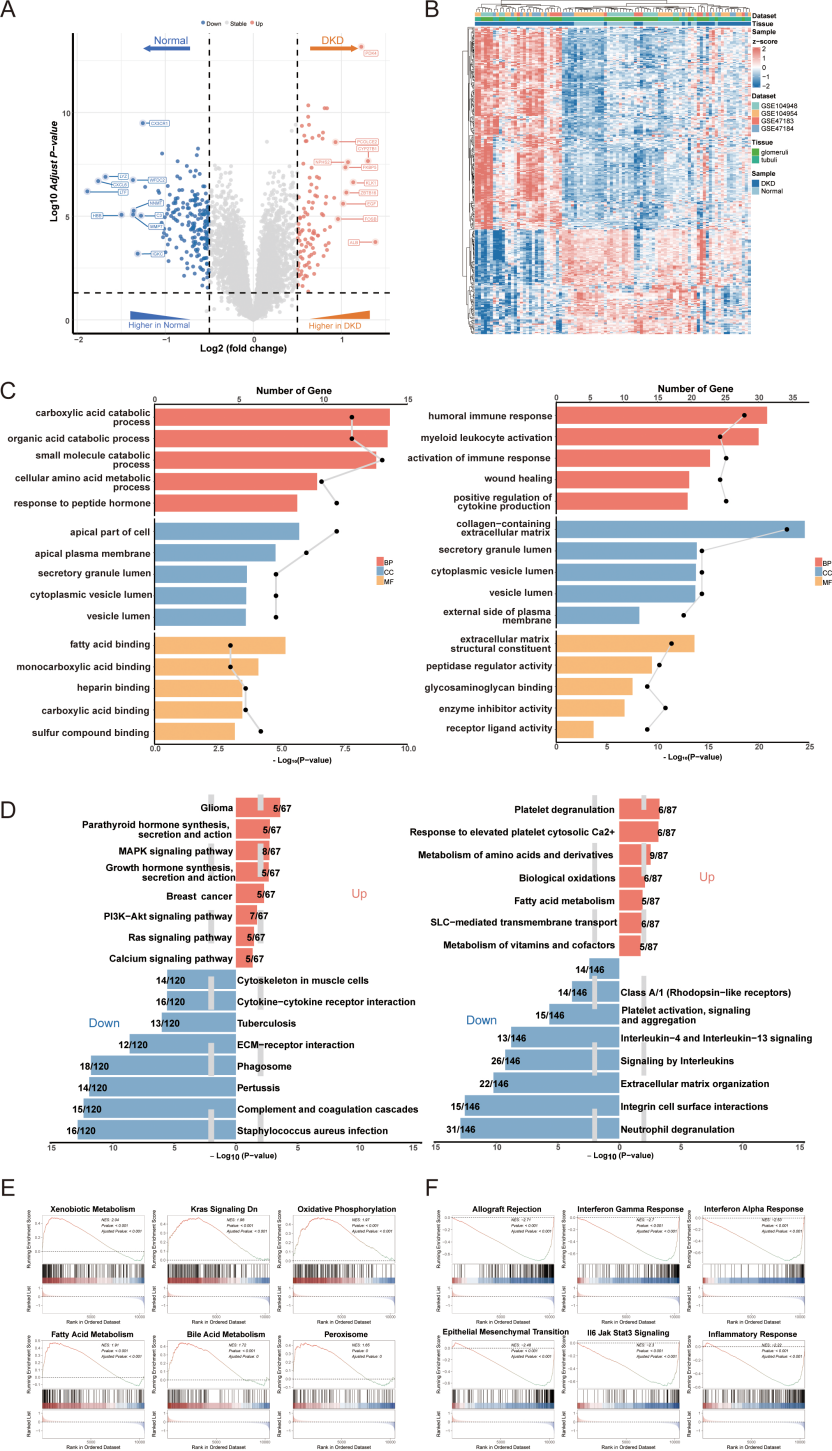
Identification of differentially expressed genes (DEGs) in DN and functional annotation**. (A)** Volcano plot of DEGs between DN and controls. **(B)** Heatmap of gene expression patterns between DN and controls. **(C)** Go enrichment analysis of upregulated DEGs and downregulated DEGs. **(D)** KEGG and REACTOME pathway enrichment analysis of DEGs in DN. **(E)** Positive GSEA enrichment of DEGs. **(F)** Negative GSEA enrichment of DEGs. DN, diabetic nephropathy; GO, gene ontology; KEGG, Kyoto encyclopedia of genes and genomes; GSEA, gene set enrichment analysis.

A total of 296 DEGs in DN were identified using thresholds of adjusted p-value < 0.05 and |log2FC| > 0.5 (**Figure 2A**, **Supplementary Table 2**). Among these, 195 DEGs were downregulated, including LYZ, CX3CR1, C3, and CXCL6, which are implicated in renal inflammation and immune cell activation. Conversely, 101 DEGs were upregulated, including KLK1, ALB, and PDK4, which play roles in fluid and electrolyte balance as well as energy metabolism in the kidney. The distribution of DEGs indicates distinct gene expression patterns between DN and controls (**Figure 2B**). To elucidate the biological mechanisms underlying DN, pathway enrichment analyses were conducted for both upregulated and downregulated DEGs. The GO enrichment results revealed increased catabolism, including carboxylic acid and cellular amino acid catabolism in DN. Additionally, adaptive immune responses such as humoral immune response, bone marrow leukocyte activation, and positive regulation of cytokine production were found to be dysregulated (**Figure 2C**, **Supplementary Table 3**). KEGG and REACTOME pathway analyses corroborated these findings, indicating excessive activation of fatty acid metabolism and dysregulation of complement and coagulation cascades (**Figure 2D**, **Supplementary Tables 3**). These pathway enrichment results suggest a state of heightened catabolism and abnormal immune response activation during the progression of DN. Furthermore, The GSEA enrichment analysis using hallmarks from the MsigDB database indicated positive enrichment in oxidative phosphorylation and peroxisomes, alongside negative enrichment in interferon gamma and alpha responses (**Figures 2E, F**, **Supplementary Table 3**). Previous literature discussed the imbalance of the oxidative-antioxidant system in DN, highlighting increased oxidative stress due to high glucose, which induces endothelial cell apoptosis and fibrosis, leading to renal histological and functional abnormalities (32, 33). However, research on the antiviral infection response in DN remains limited.

### 3.3 Evaluating key modules in weighted gene co-expression network and recognizing GRGs in DN

**Figure 3:**
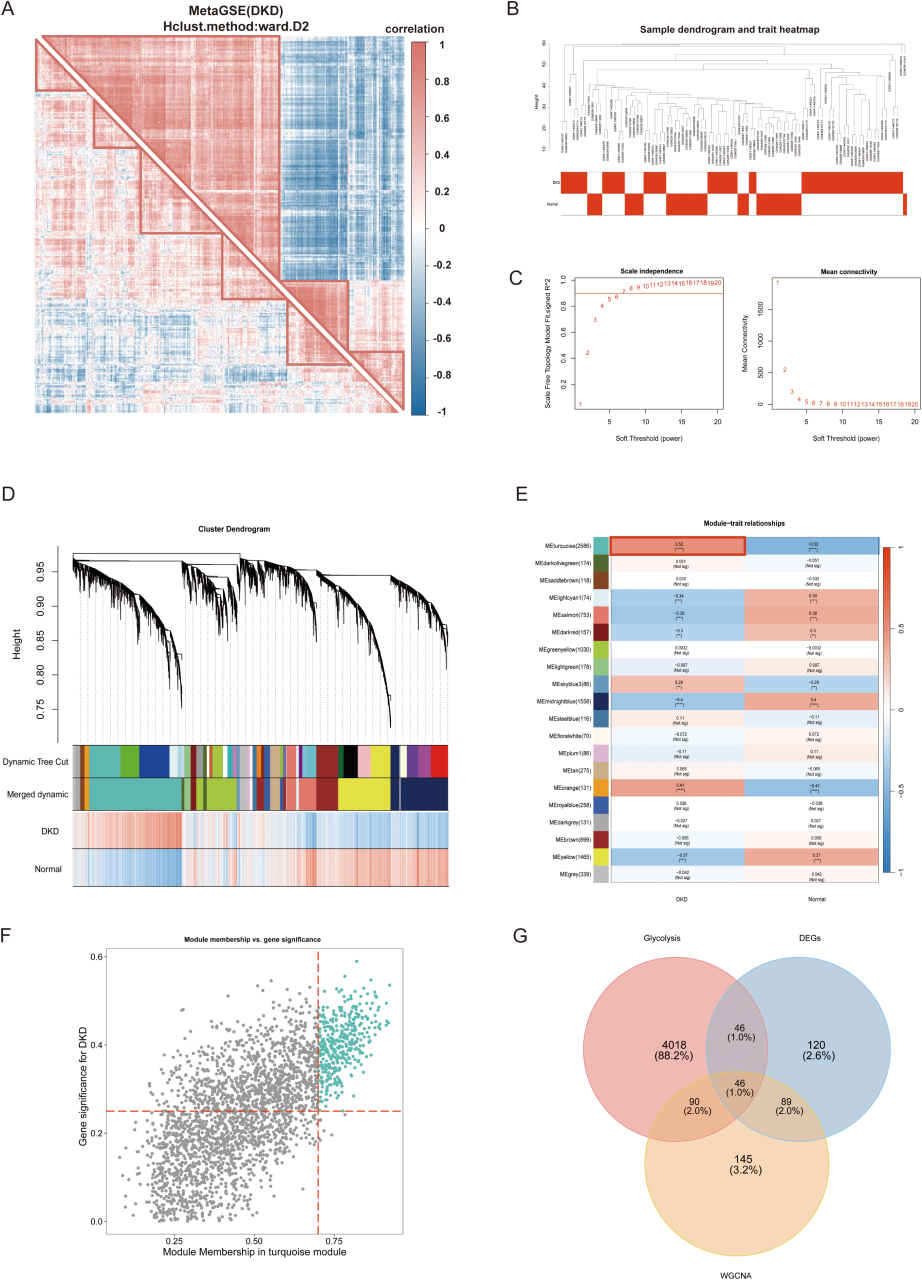
Weighted gene co-expression network analysis (WGCNA) and glycolysis-related genes (GRGs) identification. **(A)** Correlation analysis of differentially expressed genes between DNc and controls. **(B)** Samples clustering tree. **(C)** The soft threshold power and mean connectivity of WGCNA. **(D)** The cluster dendrogram. **(E)** The heatmap depicting the relationship between modules and clinical traits, specifically DN and controls. **(F)** The scatter plot between gene significance and module membership in turquoise module. **(G)** The venn diagram of the intersectiong of DEGs, turquoise module genes, and glycolysis candidate genes for screening GRGs. WGCNA, weighted gene co-expression network analysis; DN, diabetic nephropathy; GRG, glycolysis-related genes. p values were showed as: **p < 0.01, ***p < 0.001, ****p < 0.0001.

In the initial phase of this study, we identified potential differences in gene expression patterns between diabetic kidney disease with DN and control groups. Subsequently, DEGs from the MetaGSE cohort were selected for clustering and correlation analyses. The results demonstrated distinct clustering and correlations among these genes within the DN and control groups (**Figure 3A**). For example, PDK4 in DN exhibited positive correlations with APOM, C1QA, ELF3, and GPR65, which were not observed under normal conditions. This indicates that focusing solely on previously screened DEGs may be insufficient. To more comprehensively identify key genes associated with the DN phenotype, we conducted a Weighted Gene Co-expression Network Analysis (WGCNA). After hierarchical clustering of all samples, we excluded outliers (**Figure 3B**). An optimal soft threshold of power = 7 (R² = 0.9) was selected to construct a scale-free topological network (**Figure 3C**). In this study, we set the minimum module gene count to 50 and the MEDissThres to 0.3, ultimately identifying 20 co-expression modules (**Figure 3D**). Our findings revealed that the MEturquoise module exhibited the strongest positive correlation withDN in the MetaGSE cohort (cor = 0.52, p = 1.21e-07, **Figure 3E**). Furthermore, the gene significance (GS) of the MEturquoise module showed a significant correlation with module membership (MM) (cor = 0.68, p < 1e-200, **Figure 3F**). These results suggest that the genes within the MEturquoise module may possess functional relevance in DN. We screened 370 critical genes from the MEturquoise module based on the criteria GS > 0.25 and MM > 0.7 (**Supplementary Table 2**). Glycolysis-related candidate genes were compiled from relevant literature, the GeneCards (https://www.genecards.org/) (34), as well as from the HALLMARK_GLYCOLYSIS and REACTOME_GLYCOLYSIS gene sets within the MsigDB (18) (**Supplementary Table 2**). By intersecting these with the previously identified DEGs, we filtered out 46 GRGs (**Figure 3G**).

### 3.4 Construction and validation of the diagnostic signatures based on integrative machine learning

**Figure 4:**
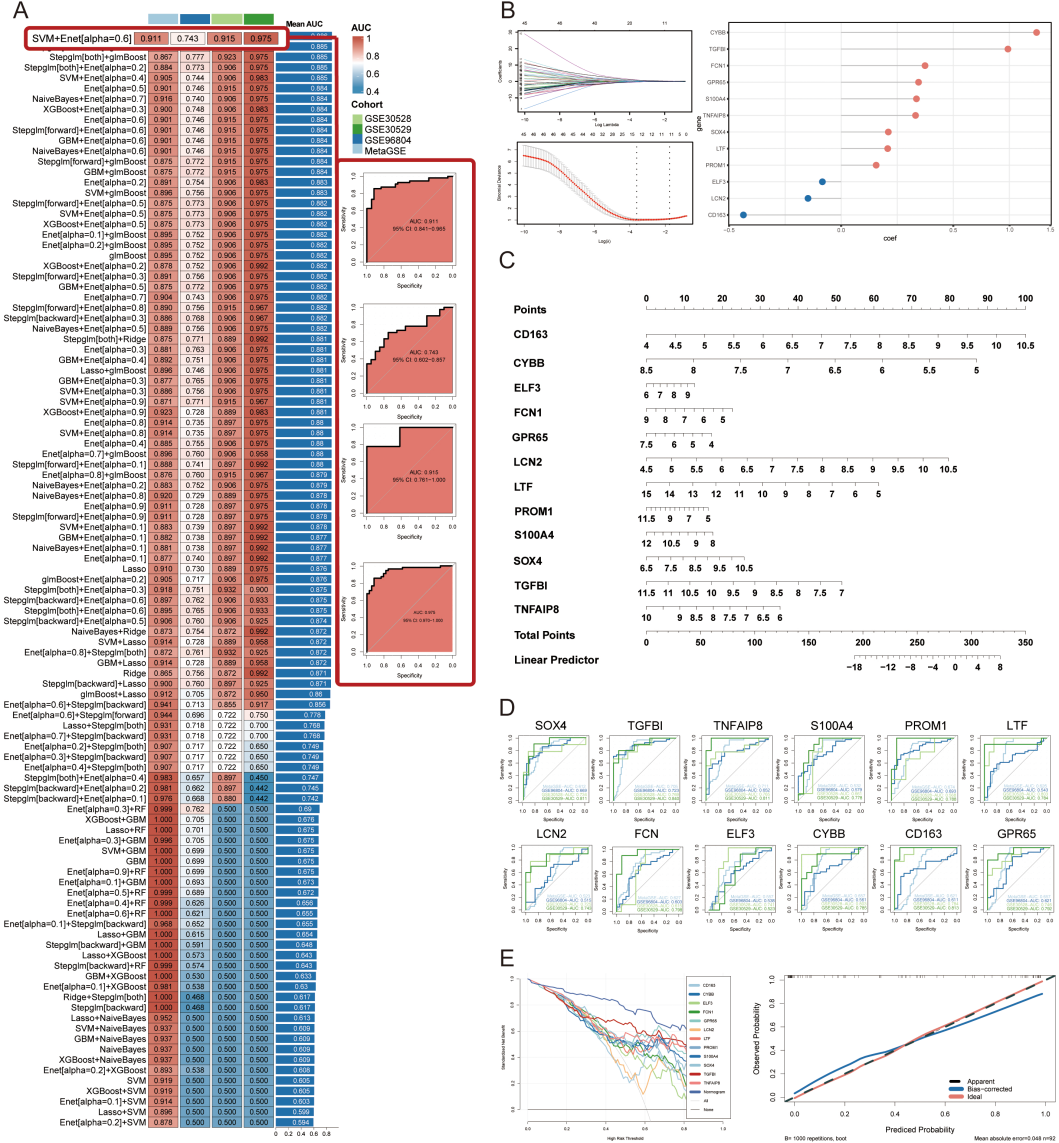
Construction and validation of diagnostic signatures by integrative machine learning. **(A)** The 108 combinations of prediction models using 10-fold cross-validation with ranked AUC index. **(B)** Visualization of elastic net regression in MetaGSE cohort and the coefficience of diagnostic signatures. The optimal lambda was retrieved when the binomial deviance reached the minimum value. **(C)** Nomogram of the 12 diagnostic signatures. **(D)** ROC plots for each diagnostic signature in internal training cohort and external validation cohorts. **(E)** Decision curve showing the net benefit by applying the nomogram and all diagnostic signatures. Calibration curve shows the predicted performance of the nomogram.

To construct diagnostic signatures based on GRGs, we employed 108 combinations of 10 machine learning algorithms for variable selection and model development. The combination of each machine learning algorithm can be referred to in **Supplementary Table 4**. The MetaGSE cohort was divided into a training set and an internal validation set at a ratio of 7:3, ensuring a balanced distribution of clinical characteristics. Within the internal training set, we conducted 10-fold cross-validation to evaluate each algorithm combination and calculate AUC values. The ranking of AUC values for all algorithms is presented in **Figure 4A** and **Supplementary Table 4**. Notably, the combination of Support Vector Machine (SVM) with Elastic Net (Enet; alpha = 0.6) demonstrated superior performance across both internal and external datasets, achieving an AUC of 0.911 (95%CI: 0.841 - 0.965) in the training/internal validation set and AUCs of 0.743 (95%CI: 0.602 - 0.857), 0.915 (95%CI: 0.761 - 1.000), and 0.975 (95%CI: 0.870 - 1.000) in the external validation sets. The AUCs and 95% Cis for all models can be found in **Supplementary Table 4**. The lower AUC in the GSE96804 dataset may be attributed to the control samples, which were normal tissues adjacent to surgically removed tumors and likely exhibited underlying transcriptional abnormalities. In summary, the SVM + Enet (alpha = 0.6) algorithm combination merged as a highly accurate predictive model. Under 10-fold cross-validation frame, we identified the optimal lambda value of 0.02732 (referred to as lambda.min, that with the smallest average error) in the SVM + Enet model. This analysis led to the identification of 12 key genes (SOX4, TGFBI, TNFAIP8, S100A4, PROM1, LTF, LCN2, FCN, ELF3, CYBB, CD163, GPR65) along with their coefficients (**Figure 4B**). Moreover, we developed a predictive nomogram for the occurrence and progression of DN assigning score points to each diagnostic signature. In the nomogram, each diagnostic signature is assigned a score point. The total score, derived from the sum of these points, predicts the risk of DN (**Figure 4C**). Previous attempts to establish predictive models related to glycolysis or DN predominantly utilized lasso regression, which tends to emphasize predictive performance over the actual state of glycolysis. To address this limitation, we used the 12 diagnostic signatures as the input gene set to construct a score model based on GRGs, named GScore. The formula is as follows: GScore = ssGSEAsocre (12 diagnostic signatures).Calibration curves confirmed the accuracy of the GScore in predicting DN (**Figure 4D**). Additionally, the decision curve analysis demonstrated that the predictive performance of the nomogram based on the 12 diagnostic signatures surpassed that of individual genes, suggesting that it could potentially benefit DN patients (**Figure 4D**). Finally, we calculated AUC values for each diagnostic signature in both the training and validation cohorts, revealing that the predictive power of individual signatures was inferior to that of the combined GScore model. This indicates that the GScore model, based on the 12 diagnostic signatures, possesses the most robust predictive capability (**Figure 4E**).

### 3.5 Immune infiltration analysis and association with diagnostic signatures

**Figure 5:**
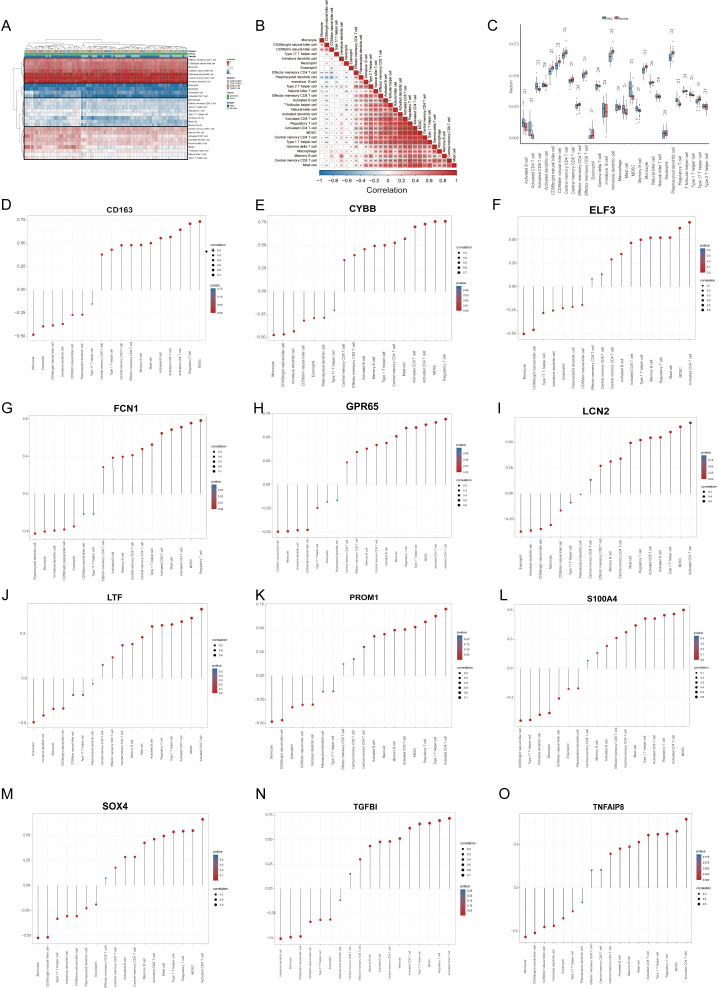
Immune infiltration analysis and association with diagnostic signatures. **(A)** Heatmap demonstrating immune cell infiltration in the DN and the control groups. **(B)** Correlations of various infiltrated immune cells **(C)** Boxplot shows infiltrated immune cell differences between DN and controls. **(D–O)** The associations between diagnostic signatures and infiltrated immune cells. DN, diabetic nephropathy. p values were showed as: *p < 0.05, **p < 0.01, ***p < 0.001, ****p < 0.0001; ns: not significant.

The ssGSEA was employed to characterize the abundance of immune cell infiltration, aiming to elucidate the immune microenvironment characteristics of DN and explore the relationship between diagnostic signatures and these characteristics. We applied z-score normalization to standardize the values of different immune cells within the range of 0 to 1 for ease of comparison and presentation. Our analysis revealed a multi-tiered infiltration pattern between DN and normal groups (**Figure 5A**). Specifically, the first-tier cell subset, represented by central memory CD4 T cells, exhibited high infiltration levels. The second-tier subset, represented by neutrophils, showed low infiltration, while the third-tier subset, represented by regulatory T cells, displayed mid-low infiltration. The fourth-tier subset, indicated by activated CD8 T cells, demonstrated mid-high infiltration levels. Subsequently, we compared the infiltration characteristics of each immune cell subset between the DN and normal groups. Activated lymphocytes, including activated B cells, activated CD4 T cells, and activated CD8 T cells, exhibited higher infiltration levels in DN relative to the normal group. Conversely, certain myeloid cells, such as monocytes and immature dendritic cells, displayed elevated infiltration levels in the normal group (**Figure 5B**). These findings suggest that myeloid cell subtypes may exist in an immunosuppressive state in DN, which aligns with previous enrichment results. **Figure 5C** illustrates the correlations among various immune cell subsets. Notably, activated CD4 T cells showed the highest positive correlation with myeloid-derived suppressor cells (cor = 0.908), indicating a strong interactive relationship between lymphocytes and myeloid cells in DN. Finally, we examined the correlations between primary infiltrating immune cells in DN and the 12 diagnostic signatures (**Figures 5D–O**).

### 3.6 Clinical risk stratification and biological features analysis based on GScore

**Figure 6:**
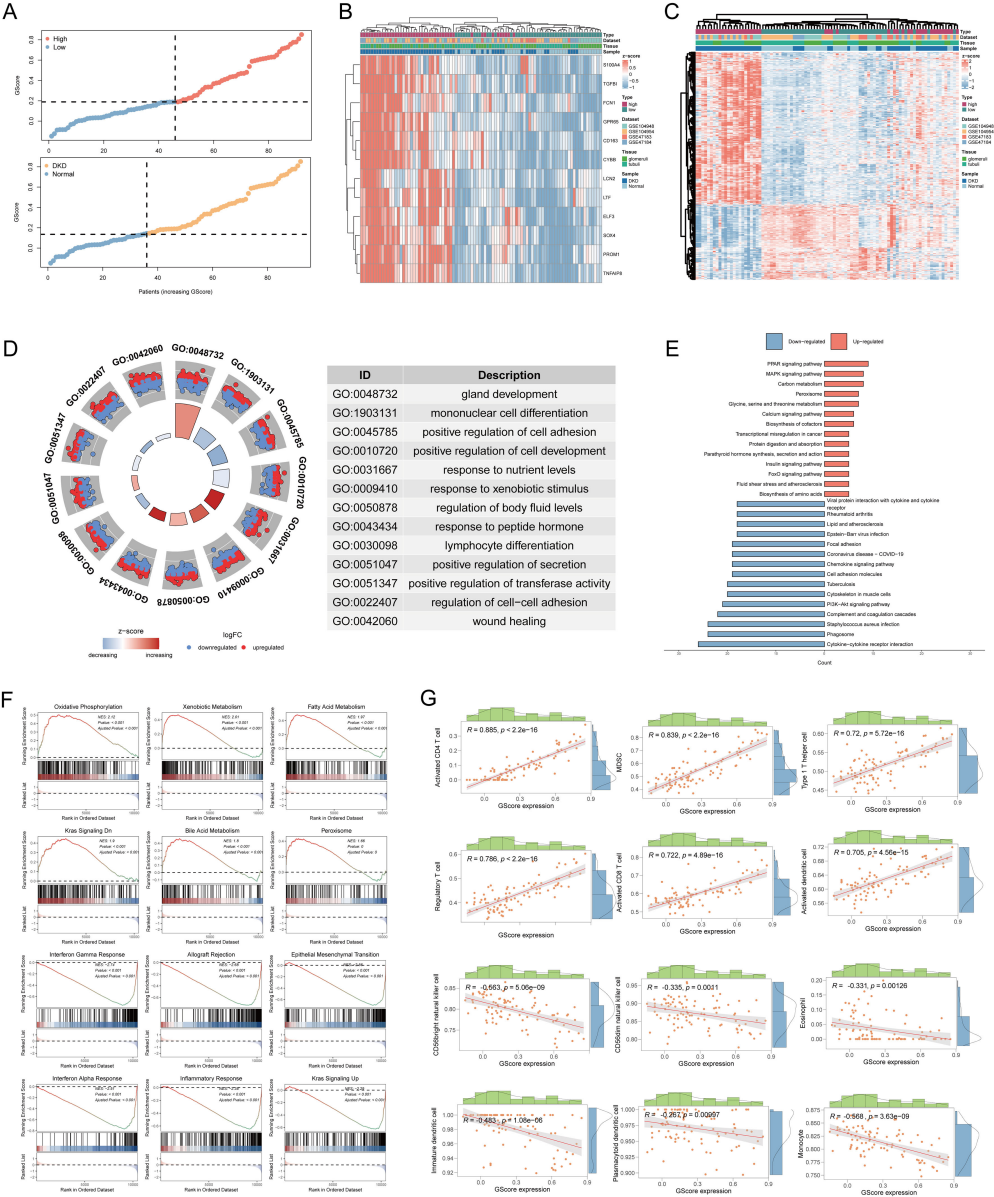
Risk stratification and biological characteristics analysis. **(A)** Distribution of risk scores and all samples. **(B)** Heatmap showing the expressions of the 12 diagnostic signatures. **(C)** Heatmap showing the differentially expressed genes between high-risk group and low-risk group. **(D)** The GO functional enrichment of differential high-risk group and low-risk group. **(E)** The KEGG functional enrichment of differential genes. **(F)** The GSEA analysis of differential expression genes. **(G)** Correlation between infiltrated immune cells and GScore. GO, gene ontology; BP, biological process; CC, cellular component; MF, molecular function; KEGG, Kyoto encyclopedia of genes and genomes; GSEA, gene set enrichment analysis.

We calculated the GScore for each sample in the MetaGSE cohort. Based on the median GScore, samples were stratified into high-risk and low-risk groups to assess the clinical stratification accuracy of the GScore. Results indicated that individuals in the high-risk group were more predisposed to DN, while those in the low-risk group were more likely to be normal (**Figure 6A**). The expression patterns of the 12 diagnostic signatures were consistent across both groups, with most signatures upregulated in the high-risk group (**Figure 6B**). Subsequently, we investigated the biological characteristics distinguishing the high-risk and low-risk groups, identifying 575 DEGs, of which 385 were downregulated and 190 were upregulated (**Figure 6C**). We performed GO and KEGG enrichment analyses based on these DEGs. Results revealed that the DEGs were primarily associated with gland development, mononuclear cell differentiation, positive regulation of cell adhesion, the insulin signaling pathway, and the PI3K-Akt signaling pathway (**Figures 6D, E**). Additionally, we conducted GSEA on the DEGs, yielding results like previous GSEA enrichments between DN and normal conditions (**Figure 6F**). Finally, we explored the relationship between GScore and immunological characteristics (**Figure 6G**). The GScore exhibited a strong positive correlation with activated CD4 T cells (cor = 0.885, p < 2.2e-16) and a negative correlation with monocytes (cor = -0.568, p = 3.63e-09).

### 3.7 Heterogeneity of GScore status in immune microenvironment at the single-cell transcriptome level

**Figure 7:**
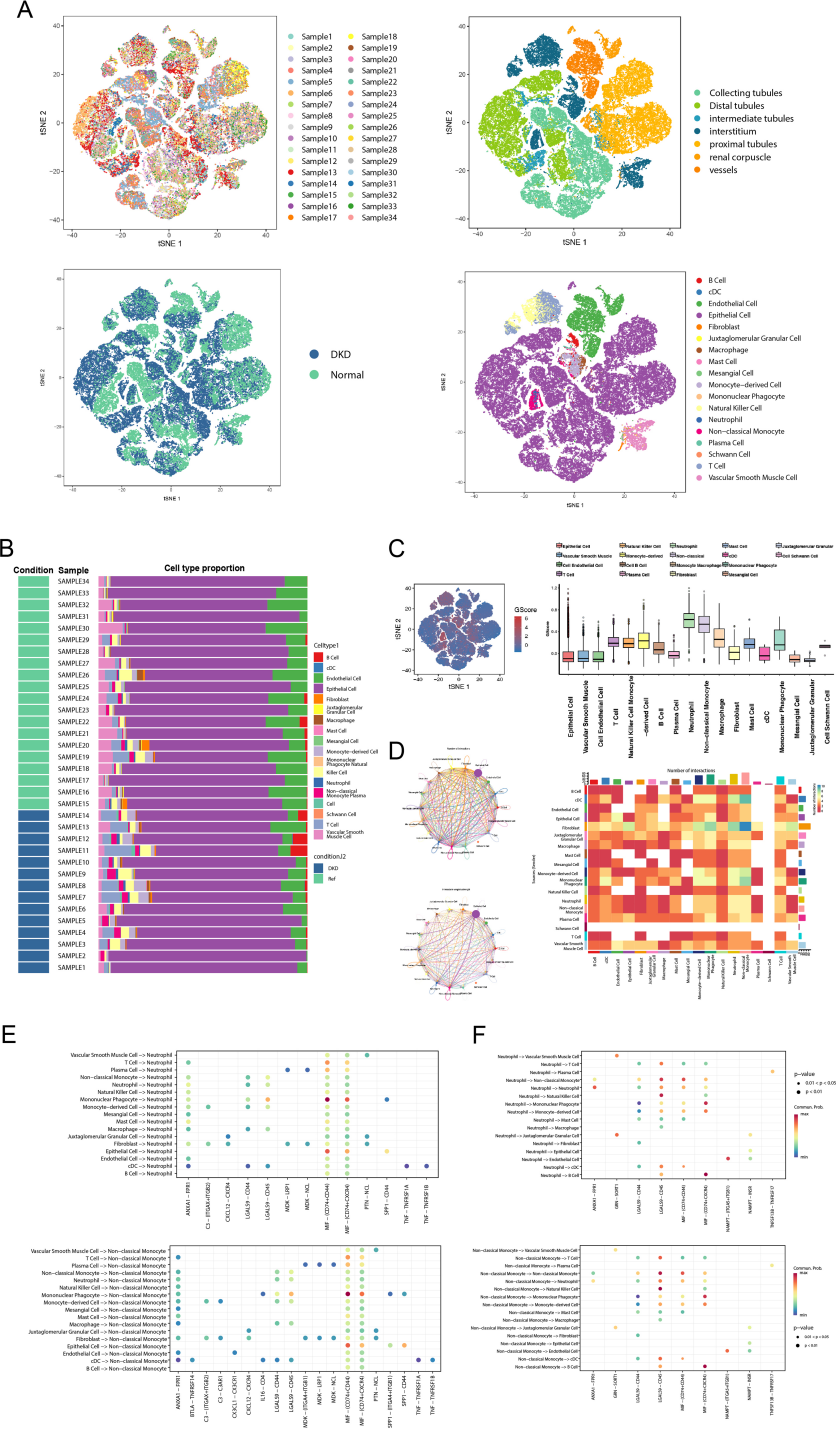
GScore characteristics in the single-cell transcriptome. **(A)** The t-SNE plot shows sample composition, tissue sources, and cell subtypes. **(B)** Stacked bar chart displaying the cell subtypes proportion of each sample. **(C)** The distribution of the GScore in all cell subtypes. **(D)** The circle plot and heatmap showing the cell communication weights and numbers of all cell subtypes. **(E)** The receptor-ligand communication weights in non-classical monocytes. **(F)** The receptor-ligand communication weights in neutrophils. t-SNE, t-Distributed Stochastic Neighbor Embedding.

To further elucidate the relationships between GScore and immune cells, we mapped the GScore onto more granular single-cell RNA sequencing data. We utilized the public dataset GSE183276, comprising 14 samples from DN patients and 20 samples from normal controls (**Figure 7A**). The tissues for single-cell sequencing were derived from various renal structures, including collecting tubules, distal tubules, intermediate tubules, interstitium, proximal tubules, renal corpuscles, and vessels (**Figure 7A**). Using established markers, we identified multiple cell types: B cells (CD79A, MS4A1), classical dendritic cells (CD11C, CLEC9A), endothelial cells (PECAM1, VWF), epithelial cells (EPCAM), fibroblasts (MME, FGF7), juxtaglomerular granular cells (REN), macrophages (CD163, CD206), mast cells (KIT, CPA3, CST3), mesangial cells (ACTA2, PDGFRB), monocyte-derived cells (CD14, CD16, CD68, CD163), mononuclear phagocytes (CD163, CD206, CD14), natural killer cells (CD16, CD56), neutrophils (CSF3R, FPR2), non-classical monocytes (CD14, CD16, CD11C, CD163), plasma cells (CD19, JCHAIN, CD27), Schwann cells (MPZ, NCAM), T cells (CD3D, CD8A), and vascular smooth muscle cells (CD36, CA4, ACKR1) (**Figure 7A**). We evaluated the proportion of each cell subtype within each sample (**Figure 7B**), revealing that lymphocytes, particularly T and B cells, were prevalent in most DN samples. To investigate the correlation of our previously developed GScore model with all cell subsets, we employed the AUCell algorithm to map the GScore of each cell type. Results indicated that neutrophils and non-classical monocytes exhibited the highest GScores (**Figure 7C**). We then quantified the strength and frequency of cell communications across all subtypes (**Figure 7D**), noting significant interaction strength and quantity for neutrophils and non-classical monocytes. These findings suggest that neutrophils and non-classical monocytes may play pivotal roles in glycolysis abnormalities associated with DN. Interactions between non-classical monocytes and neutrophils with other cell subsets were predominantly mediated through LGALS9-CD45, MIF-(CD74+CD44), and MIF-(CD74+CXCR4) pathways (**Figures 7E, F**). These results indicate specific common signaling pathways involved in the progression of DN, which have been partially explored in other systemic diseases (35–37); however, they remain understudied in the context of DN and warrant further investigation.

### 3.8 Prediction of potential therapeutic molecules

**Figure 8:**
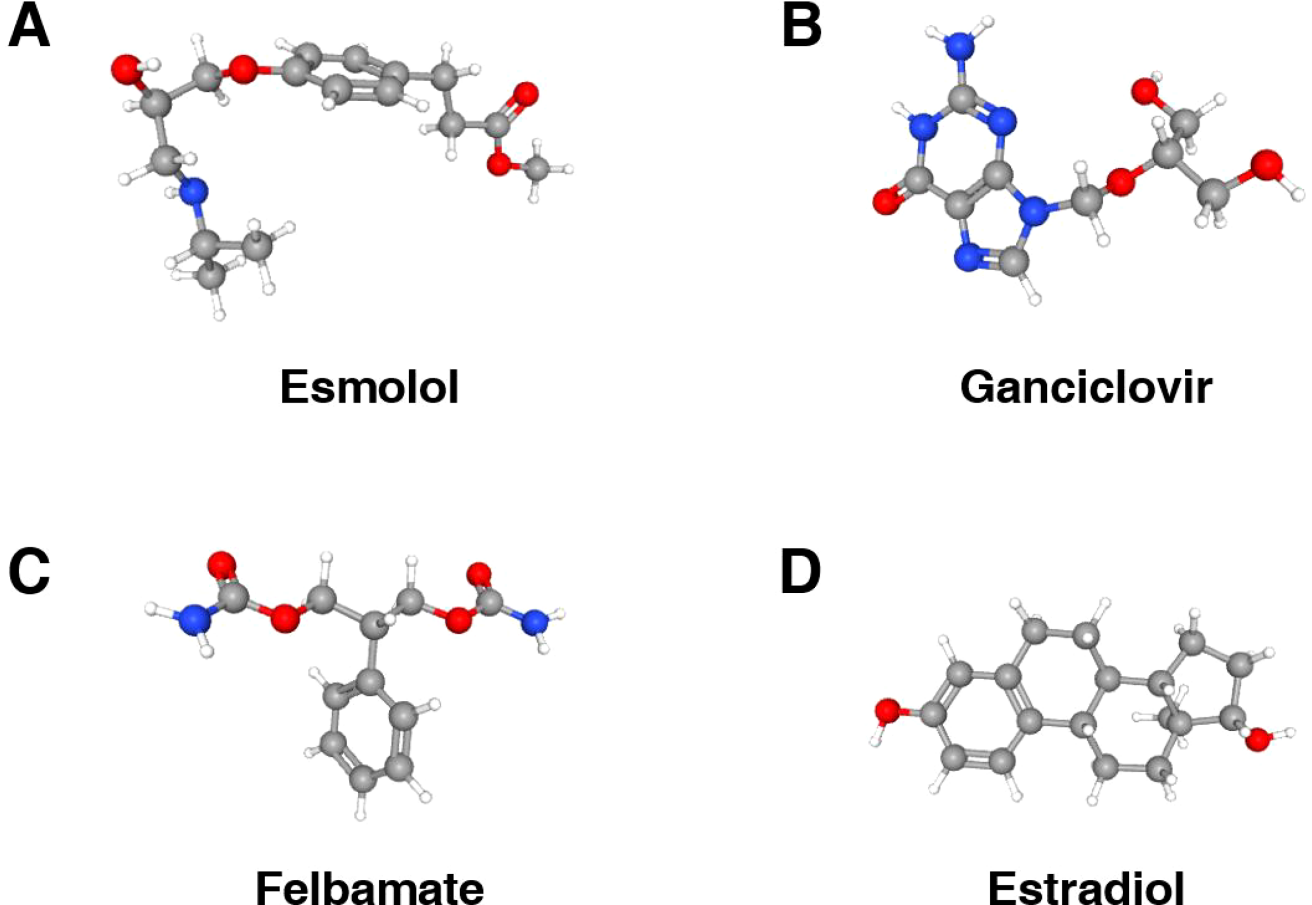
3D structures of potential therapeutic small molecule agents. **(A)** The 3D structure of esmolol. **(B)** The 3D structure of ganciclovir. **(C)** The 3D structure of felbamate. **(D)** The 3D structure of extradiol.

We identified potential therapeutic agents for the treatment and management of DN using the 12 diagnostic signatures from the cMAP database (https://clue.io/) (29). Based on the relevance scores of each agent relative to the diagnostic signatures (**Supplementary Table 5**), we selected four agents with significantly negative scores that may inhibit DN progression: esmolol (**Figure 8A**), estradiol (**Figure 8B**), ganciclovir (**Figure 8C**), and felbamate (**Figure 8D**). The 3D structural examples of these small molecules were sourced from PubChem (https://pubchem.ncbi.nlm.nih.gov/) (30). Research indicates that estradiol reduces the synthesis of angiotensin II and endothelin, thereby inhibiting renal vasoconstriction and alleviating renal inflammatory responses (38). Additionally, estradiol modulates vascular permeability by upregulating nitric oxide synthase and vascular endothelial growth factor expression, which may be beneficial in treating and preventing female diabetic nephropathy (39). However, further clinical trials are necessary to establish its specific efficacy, safety, and practicality across diverse populations.

### 3.9 Expression level of diagnostic signatures at the vitro level

**Figure 9:**
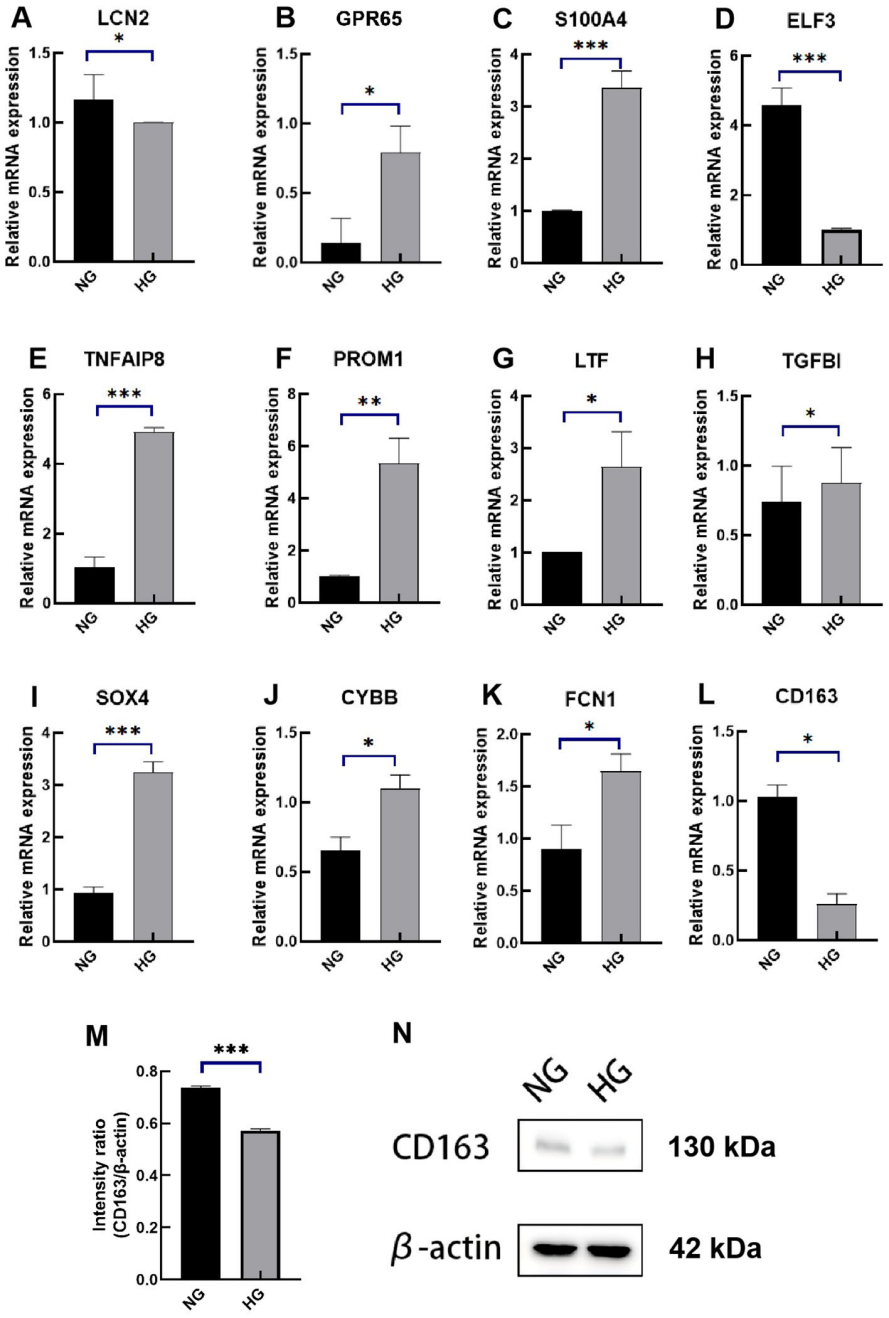
Expression levels of the 12 diagnostic signatures in vitro. **(A–L)** mRNA expression of LCN2, GPR65, S100A4, ELF3, TNFAIP8, PROM1, LTF, TGFBI, SOX4, CYBB, FCN1, CD163 by RT-qPCR. **(M)** The intensity ratio of CD163/β-action. **(N)** The WB band of CD163 in high-glucose experimental group and control group. RT-qPCR, quantitative real-time polymerase chain reaction; WB, western blotting; NG, normal group; HG, high-glucose group. p values were showed as: *p < 0.05, **p < 0.01, ***p < 0.001.

To evaluate the diagnostic value of the 12 signatures, we established a high glucose-induced HK-2 cell line, as detailed in the Methods section. Cell viability exceeded 80% at baseline, with a glucose concentration of 17.5 mM designated as the control group and 45 mM as the experimental group. Real-time polymerase chain reaction (RT-qPCR) was conducted after 48 hours of culture. The RT-qPCR results indicated that the mRNA expression levels of ELF3, LCN2, and CD163 were significantly reduced in high glucose group compared to the control group, while CYBB, FCN1, PROM1, GPR65, LTF, S100A4, SOX4, TGFB1, and TNFAIP8 showed increased expression (**Figures 9A–L**). According to the coefficients in GScore and previous single-cell analysis results, we further evaluated the expression of the key marker CD163 by Western blotting (WB). WB results demonstrated that CD163 expression was lower in the high glucose group (**Figures 9M, N**).

## 4 Discussion

Diabetic nephropathy (DN) is a chronic kidney disease resulting from diabetes mellitus (DM) and increasingly constitutes the primary cause of end-stage renal disease among the elderly in numerous countries (40). Diagnosis of DN typically occurs in patients with confirmed diabetes after excluding other primary or secondary glomerular diseases and systemic conditions. This diagnosis relies on abnormalities in the urinary albumin-to-creatinine ratio or urinary albumin excretion rate, alongside estimated glomerular filtration rate criteria (11). However, early-stage DN often presents with varying degrees of renal function decline before fulfilling these diagnostic criteria. While various pharmacological treatments are available for most DN patients, including kidney transplantation for those at end-stage, long-term management remains inadequate, with many patients needing continuous medication or regular dialysis. Early diagnosis and intervention can significantly enhance patient outcomes. Currently, clinical biomarkers are insufficient to facilitate early diagnosis or predict DN progression and prognosis. Thus, developing novel diagnostic biomarkers and identifying potential therapeutic targets are essential. Glycolysis is the metabolic pathway through which cells convert glucose into energy. In diabetic nephropathy (DN), abnormal activation or regulation of glycolysis can lead to renal damage and exacerbate disease progression. Research indicates that key glycolysis enzymes, such as pyruvate kinase M2, are significantly upregulated in DN, promoting macrophage-mediated inflammatory responses and the release of pro-inflammatory factors like TNF-α and IL-1β (41). Additionally, dysfunctional kidney cells undergo metabolic reprogramming, actively engaging in aerobic glycolysis. The resulting intermediates contribute to oxidative stress and fibrosis, further aggravating kidney injury (42). These findings suggest that GRGs may serve as potential diagnostic markers for DN. However, the underlying biological mechanisms of GRGs remain poorly understood, necessitating further investigation into their diagnostic efficacy and impact on disease progression. In this study, we conducted a comprehensive analysis of bulk transcriptome data from diabetic nephropathy (DN) and normal kidney tissues. We identified 296 DEGs between DN and control samples and performed functional and pathway enrichment analyses. Utilizing a large training dataset, we selected twelve GRGs—CD163, CYBB, ELF3, FCN1, PROM1, GPR65, LCN2, LTF, S100A4, SOX4, TGFB1, TNFAIP8—through 108 combinations of ten machine learning algorithms, including stepglm. We subsequently developed a scoring system, termed GScore, using the algorithm combination SVM + Enet (α = 0.6), which yielded the highest combined AUC value. The model was validated on three independent external datasets, confirming its robust predictive performance and stability.

Previous studies have partially illuminated the roles of some of these GRGs in DN progression. Specifically, CD163, a membrane receptor primarily expressed on monocytes and M2 macrophages, showed a significant positive correlation with glomerular filtration rate, contrasting early clinical features of DN characterized by glomerular ultrafiltration (43). More and preclinical studies are needed to explore whether it can be used as an early diagnostic signature. In our cellular experiments, CD163 expression was found to be reduced in DN models. Additionally, restoring CD163+ M2 macrophages in STZ-induced DN rats improved renal function, suggesting that targeting CD163 may represent a promising therapeutic strategy for managing long-term complications of type 2 DM (44). The activation of TGF-β/Smad3 signaling in DN patients promotes the secretion of ELF3-containing urinary exosomes from podocytes, correlating closely with glomerular filtration rate decline (45). This positions urinary exosomal ELF3 protein levels as potential non-invasive biomarkers for early podocyte injury in DN. The expression level of PROM1 shows dynamic changes during DN progression. Meanwhile, PROM1 expression exhibits dynamic changes throughout DN progression; it increases in proximal renal tubular cells as DN advances, indicating a protective role that reflects the ongoing processes of renal tissue injury and repair, although its molecular mechanisms remain unexplored (46). TNFAIP8, a less-studied regulator of nuclear immune homeostasis, demonstrated significantly elevated expression in DN cell lines. Its expression correlates directly with mesangial cell proliferation, potentially mediated by NADPH oxidase pathways. Deeper molecular investigations are needed to elucidate the mechanisms linking TNFAIP8 to diabetic kidney injury (47). Similarly, SOX4 has been shown to induce mesangial cell proliferation and fibrotic phenotypes via the LncRNA SNHG14/miR-30e-5p/SOX4 axis (48). Additionally, we observed significant upregulation of FCN1, GPR65, S100A4, and TGFBI in DN cell lines, although high-quality studies investigating their molecular mechanisms in DN progression are limited (49). It is worth highlighting that most current published studies have some oversight. Most of them only consider AUCs when selecting models, but ignore the importance of 95% Cis, especially when the AUCs of several models are similar. Choosing features for modeling also requires careful consideration. Selecting too many features may lead to overfitting. Too few features may result in low specificity. The less well studied features in certain diseases may lead to distortion. The application of GScore allowed for effective risk stratification of participants in the training set, successfully distinguishing DN patients from controls. These findings provide valuable insights for the early screening of DN in clinical practice. Recently, several studies have focused on developing predictive models for diabetic nephropathy (DN). One study constructed a signature based on four core cell death genes, achieving an AUC of 0.929. In contrast to our model, this study exclusively utilized samples from glomeruli and did not include tubular tissue (49). Given that most DN patients also suffer from hypertension and hyperlipidemia, there is a notable lack of relevant differential diagnostic signatures, underscoring the clinical significance of addressing renal function impairment in diabetic patients, particularly those with comorbid hypertension. In immune infiltration analysis, we observed a significant increase in central memory CD4 T cells, dendritic cells, monocytes, M1 and M2 macrophages, and neutrophils in patients with DN. A similar trend was noted in the single-cell RNA transcript profiles, indicating abnormal overactivation of myeloid cells within the DN immune microenvironment. This imbalance between myeloid cell and lymphocyte activation may contribute to chronic renal tissue inflammation in DN. The abundance of M1 macrophages is associated with inflammatory responses and programmed cell death in DN. The interaction between Notch signaling and NF-κB signaling promotes M1 macrophage polarization. Hyperpolarized M1 macrophages secrete pro-inflammatory factors such as IL-4, IL-10, inducible nitric oxide synthase, and reactive oxygen species, which exacerbate extracellular matrix secretion, oxidative stress, and necroptosis in renal intrinsic cells (50). In contrast, M2 macrophages, known for their anti-inflammatory properties, play a role in renal tissue reconstruction post-injury (51). Notably, neutrophils and monocytes emerged as major contributors to GScore in the DN immune microenvironment. Previous studies have shown that neutrophils overdeposited in the glomerulus release extracellular traps that can induce glomerular endothelial pyroptosis (52). Monocytes, which represent a poorly differentiated pre-myeloid cell population, may alleviate DN symptoms through regulated differentiation. For example, the A2B adenosine receptor antagonist MRS1754 has been shown to reduce the expression of chemokine chemoattractants and adhesion genes in glomerular monocytes/macrophages, leading to alterations in the M1/M2 macrophage ratio, enhanced macrophage-myofibroblast transition, and reduced fibrosis and inflammation in DN (53). In summary, our findings provide initial insights into the characteristics of immune cell infiltration within the DN immune microenvironment, suggesting that various immune cell subtypes, particularly myeloid cells, play crucial roles in DN progression. This highlights potential avenues for further research on therapeutic strategies. There are several limitations that warrant acknowledgment. Although we evaluated and validated the GScore using a substantial dataset, large-scale prospective clinical trials are essential to further confirm its diagnostic and predictive capabilities. Currently, available transcriptome data for DN primarily derives from kidney tissue, whereas blood samples, which are more accessible clinically, should also be included. Thus, incorporating data from diverse sources is necessary to assess the generalizability and practicality of the GScore. Furthermore, none of the seven datasets used in this study included critical clinical information such as age, duration of diabetes, blood pressure, or blood glucose levels. These indicators are vital for determining the presence of DN risk factors. Future studies should aim to integrate clinical data with transcriptional profiles to better elucidate patients’ stratified clinical characteristics. Additionally, careful consideration of terminology used to label sample types is required. The external validation set GSE96804 exhibited moderate performance, potentially influenced by the fact that control samples were sourced from cancer patients. It is crucial to determine whether samples from individuals with underlying conditions can be classified as normal or control subjects based on the research context. While this study included experimental validation in cell models to support future investigations, further *in vitro* and *in vivo* experiments are needed to comprehensively explore the impact of these diagnostic signatures on DN progression. In conclusion, future research should focus on more extensive experimental designs, rigorous sample criteria, precise molecular investigations, and comprehensive clinical data to systematically elucidate the biological roles of GRGs in DN progression.

## 5 Conclusion

In this study, we conducted 108 combinations of machine learning algorithms to identify 12 diagnostic signatures: CD163, CYBB, ELF3, FCN1, PROM1, GPR65, LCN2, LTF, S100A4, SOX4, TGFB1, TNFAIP8. The GScore model, based on these genes, effectively stratified clinical risk and identified patients with DN. Utilizing single-cell RNA sequencing data and online drug databases, we predicted cell subsets that may influence the DN microenvironment along with potential targeted therapies. Notably, the expression levels of these signatures significantly differed *in vitro*. These findings suggest that the GScore scoring model has the potential to serve as an essential prediction and treatment decision support system for DN.

## Data Availability

The datasets presented in this study can be found in online repositories. The names of the repository/repositories and accession number(s) can be found in the article/**Supplementary Material**.

## Supplementary material

Supplementary Figure 1 Data preprocessing. **(A)** Distribution of MetaGSE before data normalization and batch correction. **(B)** Distribution of MetaGSE data after data normalization and batch correction. **(C)** The PCA plot shows data distribution before data normalization and batch correction in MetaGSE. **(D)** The PCA plot shows data distribution after data normalization and batch correction in MetaGSE.

Supplementary Table 1 Detailed datasets information.

Supplementary Table 2 Differentially expressed genes, glycolysis-related genes, and hub genes in WGCNA.

Supplementary Table 3 Results of GO/KEGG/REACTOME/GSEA enrichment.

Supplementary Table 4 The key parameters and results of machine learning frameworks.

Supplementary Table 5 Results of screening for potential agents in cMAP.
