# Supplementary figures and images for: Identification and validation of glycolysis-related diagnostic signatures in diabetic nephropathy: a study based on integrative machine learning and single-cell sequence
Source: Front Immunol. 2025 Jan 23;15:1427626. doi: 10.3389/fimmu.2024.1427626 (PMC11798943; doi:10.3389/fimmu.2024.1427626)

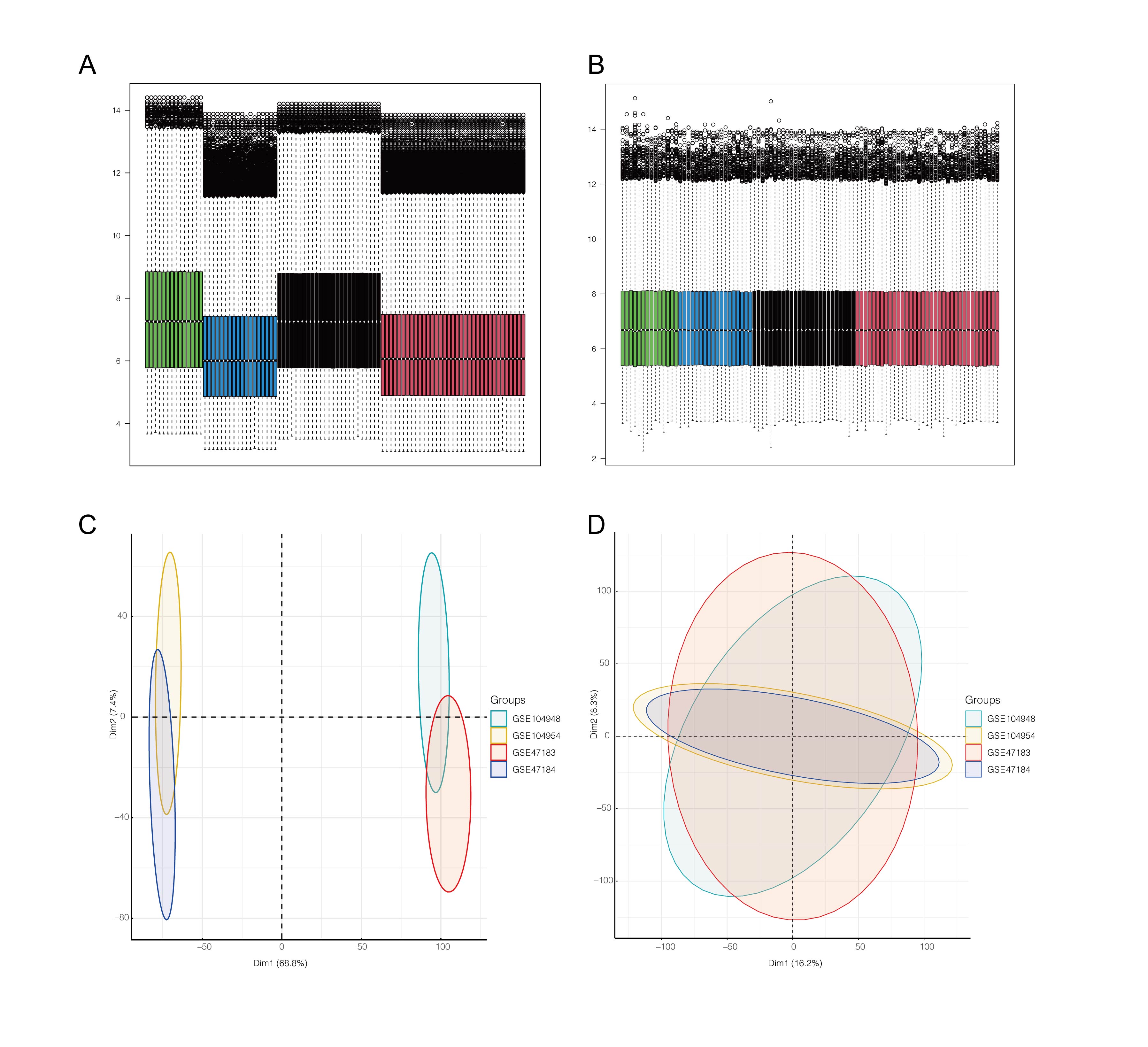

Supplement: Supplementary Figure 1 — Data preprocessing. (A) Distribution of MetaGSE before data normalization and batch correction. (B) Distribution of MetaGSE data after data normalization and batch correction. (C) The PCA plot shows data distribution before data normalization and batch correction in MetaGSE. (D) The PCA plot shows data distribution after data normalization and batch correction in MetaGSE. [file Image1.jpeg]
